# Reaction of complement factors and proteasomes in experimental encephalitis

**DOI:** 10.1007/s13365-016-0500-1

**Published:** 2016-12-02

**Authors:** Stefan Lange, Tomas Bergström, Ewa Johansson, Merna Oshalim, Ivar Lönnroth

**Affiliations:** 1 Department of Infectious Diseases, Institute of Biomedicine, Gothenburg University, P.O.B 420, S-40530 Gothenburg, Sweden; 2 Clinical Microbiology, Sahlgrens University Hospital, P.O.B, S-40530 Gothenburg, Sweden

**Keywords:** Herpes simplex virus type 1, Encephalitis, Complement factor, Proteasome, Compleasome

## Abstract

Herpes simplex virus type 1 (HSV-1) encephalitis causes a deleterious inflammation and elevated intracranial pressure. As a step towards examining the origin of the inflammation, we here report the response of circulating proteasomes and complement factors in blood and cerebrospinal fluid (CSF) in rats infected with HSV-1. Infection was via the nasal route, with 1.1 × 10^4^ plaque-forming units of HSV-1 strain 2762 given in one or both nostrils. A sandwich enzyme-linked immunosorbent assay was used to study the level of 26S proteasomes and their complex formation with complement factors 3 and 4. HSV-1 infection in the rat causes a complex formation between complement factors and proteasomes, which we designate compleasomes. In the first experiment, with HSV-1 given in both nostrils, compleasomes containing complement factors 3 and 4 increased significantly in both blood plasma and CSF. The concentration of proteasomes in plasma was similar in controls and infected rats (320 ± 163 vs. 333 ± 125 ng/ml). In the second experiment, with HSV-1 given in one nostril, CSF levels were 1 ± 1 ng/ml in controls and 56 ± 22 ng/ml in the HSV-1 group, whereas the total protein concentration in CSF remained the same in the two groups. The compleasome response was limited to CSF, with a highly significant difference between infected rats and controls (*n* = 11, *p* < 0.001). It was possible to mimic the reaction between proteasomes and complements 3 and 4 in vitro in the presence of ATP.

## Introduction

Herpes simplex virus type 1 (HSV-1), a usually benign virus that commonly causes recurrent oral lesions, may occasionally induce a necrotizing encephalitis focused on the frontotemporal regions of the brain. The pathogenetic factors responsible for HSV-1 encephalitis (HSE) in adults are obscure, and although antiviral treatment with acyclovir has significantly reduced the mortality (Sköldenberg et al. 1984), most patients suffer from neurological sequels at follow-up (Raschilas et al. 2002, Hjalmarsson et al. 2007). A prolonged intrathecal inflammatory response including activation of the complement system has been documented in HSE patients (Aurelius et al. 1993, Aurelius et al. 1994, Eriksson et al. 2016).

The ubiquitin-proteasome pathway degrades defective and foreign proteins and plays an important role in a wide variety of cell functions including antigen processing, transcription regulation, cell cycle regulation, and DNA repair. This system is manipulated by a variety of viruses, including HSV-1 (Gao and Luo 2006). The ICP0 protein expressed by HSV-1 is a ubiquitin ligase which promotes the spread of the virus by degrading various cellular proteins (Boutell and Everett 2013). Most proteasomes are located intracellularly, but circulating proteasomes have also been found extracellularly in blood and other body fluids including cerebrospinal fluid (CSF) (Sixt and Dahlmann 2008, Mueller et al. 2012).

We have previously shown that a peptide derived from the proteasome subunit antisecretory factor (AF) abolishes sickness and death in rats subjected to experimental HSE (Jennische et al. 2008). This counteraction of the lethal encephalitis is most likely accomplished by lowering the intracranial pressure. Diet-induced antisecretory factor has also been shown to prevent intracranial hypertension (Johansson et al. 2013). In order to reveal the mechanism behind this effect, we isolated a precursor of the antisecretory factor in blood, consisting of a complex of circulating proteasomes and complement factor 3 (C3) (Lonnroth et al. 2016). During formation of the complex, C3 was split into C3c, which changed the proteasome conformation to expose the hidden antisecretory peptide.

In the present study, we found that HSV-1 is able to induce similar complexes of proteasomes and complement factors 3 and 4 (C3 and C4). However, no hydrolyses of C3 or exposure of AF occurred in this complex. As a general designation of the complex between complement factors and proteasomes, we here introduce the term “compleasome”.

## Materials and methods

### General reagents

Monoclonal antibody against AF/RPN10 was produced as previously described (Johansson et al. 2009). Monoclonal mouse IgG antibodies against proteasome LMP2 (20Sβ1i), 20Sα6, ubiquitin and the proteasome 26S and 20S proteins were obtained from Enzo Life Sciences Inc. (www.enzolifesciences.com). Polyclonal antibody against proteasome 19S subunit was produced as previously described (Lonnroth et al. 2016); polyclonal antibodies against C3 and C4 were obtained from Dako (www.dako.com, items A0062 and Q0369). Secondary antibodies, alkaline phosphatase conjugated goat anti-rabbit IgG and goat anti-mouse IgM were obtained from Jackson ImmunoResearch Laboratories, Inc.

### Animal model

Male Sprague-Dawley rats with body weight 250 ± 20 g (Nova-SCB, Sollentuna, Sweden) were used. The regional ethical committee of the University of Gothenburg approved the study protocol, and all experiments were performed in accordance with the guidelines for animal experiments (EC Directive 86/609/EEC). The rats were allowed a week for general adaptation in their cages before the experiments and had free access to water and pelleted food during the experiments. The temperature and ventilation in the animal quarters were monitored according to standard procedures. The animals were infected with HSV-1 strain 2762, originally isolated from the brain of a patient with HSE, in the nostril under isoflurane anaesthesia as previously described (Jennische et al. 2015). In the first experiment, a 25 μl dose containing 1.1 × 10^4^ plaque-forming units of HSV-1 was given in each nostril to 6 rats; 5 controls receiving phosphate buffered saline (PBS; pH 7.2) were used. In the second experiment, one dose of 1.1 × 10^4^ plaque-forming units was given in the right nostril to 11 rats (12 controls receiving PBS were used). The animals were sacrificed at day 5 before development of clinical symptoms as elaborated in previous experiments (Jennische et al. 2015). For ethical reasons, the extent of the experiment could not exceed day 5, at which time the animals began to show symptoms of neurological dysfunction, i.e. repetitive, stereotypic movements and motor instability.

### Blood and CSF sampling

The rats were brought to deep anaesthesia with isoflurane (Forane, Baxter, Deerfield, IL) before the sampling took place.

#### Blood

The heart was punctured from the right thoracic cavity with an injection needle (OD 1.22 mm, length 60 mm). Following this, 10–12 ml of blood was drawn during 2–3 min and immediately mixed with heparin in order to avoid coagulation. The blood was centrifuged at 1200×*g* for 15 min, and then the plasma was mixed with an equal volume of citrate buffer (glucose 0.11 M, tri-sodium-citrate 0.03 M and sodium chloride 0.07 M; pH 6.1). Each sample was subsequently divided into 2 ml aliquots and kept at −20 °C until use. No sample was stored frozen for more than 4 weeks.

#### CSF

After termination of the rat by removal of the heart, the rat was placed on its abdomen and the neck muscles were laterally dissected to expose the midline of the dura over the cisterna magna. This line was used as a marker for puncture in order to reach CSF in the underlying cisterna magna, using a neoflon i.v. cannula (OD 0.6 mm, length 19 mm). After dura puncture, the needle was inserted a further 3–5 mm and then removed from the surrounding plastic cannula. This allowed 50–200 μl of CSF to be aspired from the cisterna magna without being contaminated with blood. The CSF was centrifuged at 1200×*g* for 5 min and the supernatant subsequently diluted with an equal volume of the citrate buffer (pH 6.1). Each sample was kept at −20 °C until use.

### Compleasome ELISA

A sandwich enzyme-linked immunosorbent assay (ELISA) for detection of compleasomes (proteasome/complement complexes) in blood plasma and liquor was performed as previously described (Lonnroth et al. 2016). Monoclonal antibodies (mab) against proteasome proteins subunits, or PBS as control were coated on a Nunc 96-well MaxiSorp polyvinyl plate (Sigma-Aldrich) overnight (dilution 1:2000 except mab AF1 which was diluted 1:400). After blocking with 0.2% bovine serum albumin (BSA) at 37 °C for 45 min, plasma and CSF samples were titrated in PBS with 0.2% BSA and 0.05% Tween 20, and shaken for 1 h. A polyclonal rabbit antibody (anti-C3 or anti-C4) at 1:2000 dilution was applied as detecting antibody. An anti-rabbit-alkaline phosphatase (AP) secondary antibody was applied after 30 min of incubation, and AP substrate was added after a further 30 min. Absorbance was read at 405 nm in a photometer, and the difference between antibody-coated samples and controls (PBS) was estimated.

### Proteasome ELISA

A sandwich ELISA detection of 26S proteasomes was performed as previously described (Lonnroth et al. 2016). Since the proteasome proteins in mammals are extremely conserved, the antibodies against the human and rat proteins cross-react. Capturing antibodies against 20Sα6 diluted 1:1000 were coated on a Nunc 96-well MaxiSorp, and the polyclonal anti-19S proteasome subunit, diluted 1:400, was used as detecting antibody. 26S proteasome (Enzo Life Sciences) was used as reference and rabbit pre-immune serum as control. The same protocol was used as previously described for compleasome ELISA.

### In vitro experiments

Proteasomes together with C3 and C4 were incubated in PBS containing 1 mM ATP (Sigma-Aldrich) at 37 °C for 3 h. Three experiments were performed with similar results. After incubation, the samples were frozen and kept at −20 °C until testing with compleasome ELISA.

### Protein determination

The protein content in CSF was determined with the Pierce BCA protein assay kit (Thermo Scientific) using a NanoDrop photometer from Saveen Werner.

### Statistics

Data are presented as mean ± SEM. Statistical significance of difference (*p* value) for two means was assessed using an unpaired Student’s *t* test, and *p* < 0.05 was considered significant.

## Results and discussion

### Two-sided infection

**Fig. 1:**
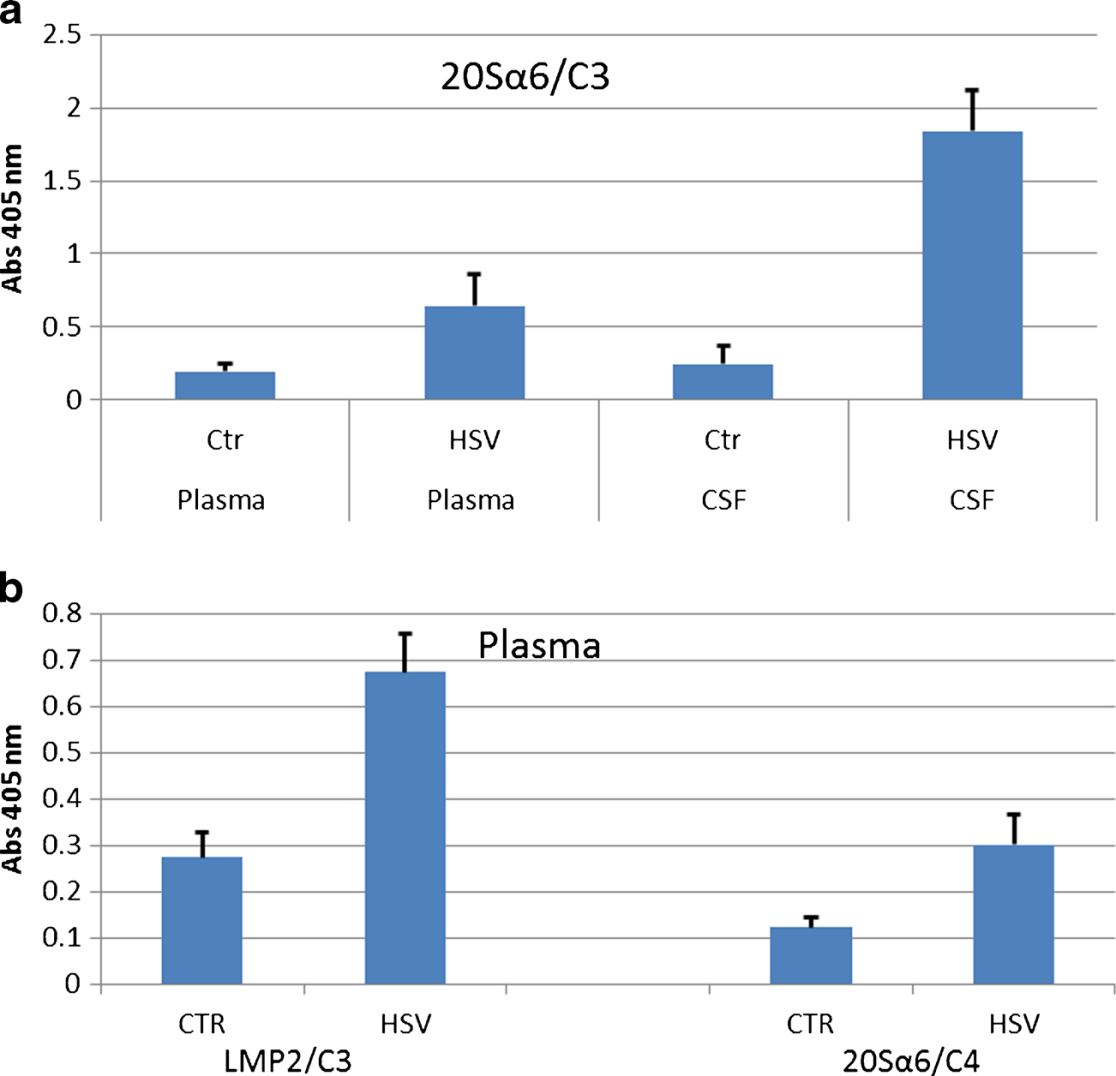
Rats after 5 days of intranasal challenge with herpes virus type 1 (HSV-1, *n* = 6) or phosphate-buffered saline as control (CTR, *n* = 5). Sandwich enzyme-linked immunosorbent assay showing compleasome complexes using monoclonal antibodies (mab) against proteasomes as catching antibody and polyclonal antibodies (pcl) against complement factors 3 and 4 (C3 and C4) as detecting antibodies. **a** Compleasomes in plasma and liquor detected with mab against proteasome subunits 20Sα6 and pcl against C3. The infected animals had significantly higher values than controls (*p* < 0.05 in plasma, *p* < 0.01 in CSF). **b** Plasma samples, mab against proteasome subunits LMP2 and 20Sα6 versus pcl against C3 or C4 as detecting antibody (HSV vs. controls *p* < 0.01 in both cases)

In the first experiment, the rats were infected with HSV-1 in both nostrils, causing a systemic infection. The experiment was ended at day 5, at which time the animals began to show symptoms of neurological dysfunction, but before the appearance of severe clinical symptoms of encephalitis. Preliminary experiments on rats infected with HSV-1 showed that an aggregation of proteasomes and C3 into compleasomes occurred at day 5 but not at day 4. Both blood plasma and CSF showed a significant increase in compleasomes when measured with antibodies against the proteasome subunit 20Sα6 and C3 (Fig. 1a). The response at day 5 was more pronounced in CSF than in blood, the ratios between test and control being 7.4 (*p* < 0.01) and 3.3 (*p* < 0.05), respectively. The monoclonal antibody against the exposed proteasome subunits LMP2 also revealed a pronounced compleasome response in plasma (*p* < 0.01, Fig. 1b). In contrast, antibodies against the hidden proteasome subunit AF1/Rpn10 or the non-proteasome protein ubiquitin gave no significant response (not shown). This suggests that the compleasome is intact and involves neither antisecretory factor nor ubiquitin.

As shown in Fig. 1b, C4-containing compleasomes were also increased in blood (*p* < 0.05). C3 and C4 are able to form complexes with each other, and such a complex might occur in the compleasome. The concentration of circulating proteasomes in blood plasma was not changed, being 320 ± 163 ng/ml in controls and 333 ± 125 ng/ml in infected rats. This is in the same range as the proteasome concentration in human plasma (Sixt and Dahlmann 2008).

### One-sided infection

In the second experiment, the rats were infected with HSV-1 in only one nostril. During this condition, the compleasome response was seen in CSF but no response was achieved in blood. The protein concentration in CSF was similar in the controls and the HSV-1 group, being 1.10 ± 0.25 and 1.32 ± 0.14 mg/ml, respectively. There thus seems not to have been any general leakage of proteins from blood to CSF in the infected animals. There were only barely detectable levels of proteasomes in CSF of the control rats (1 ± 1 ng/ml), whereas the HSV-1 group contained a relatively high level (56 ± 22 ng/ml). The significant rise of proteasomes must therefore have been caused by the HSV-1 infection, and most of the proteasomes might be bound in the compleasomes. The level of proteasomes in the infected animals was similar to that found by Mueller et al. (2012) in CSF of patients treated for spinal canal stenosis. Those authors also found no correlation between the content of proteasomes and interleukin 6, indicating that proteasomes might be formed in CSF without being initiated by an inflammatory reaction.

In previous experiments on the same rat model HSV-1 was infected via one nostril (Jennische et al. 2015), the virions and inflammation were shown to be initially localized exclusively in one brain half before spreading via the olfactory bulb to the other half. It is clear that the virions cause a local infection under these conditions, in contrast to the systemic infection achieved after administration in both nostrils.

**Fig. 2:**
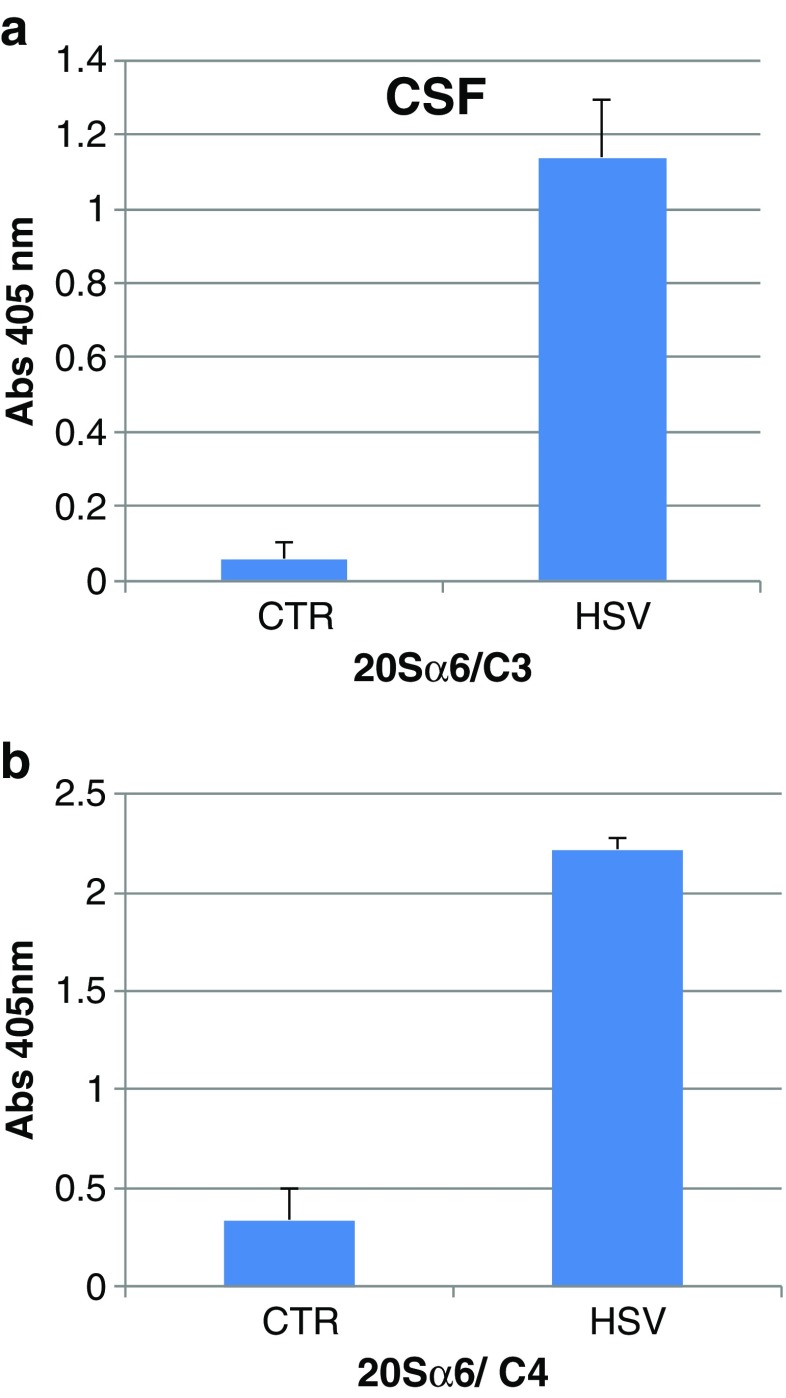
Cerebrospinal fluid (*CSF*) of rats after 5 days of challenge with herpes virus type 1 (HSV-1, *n* = 11) or phosphate buffered saline as control (CTR, *n* = 12). Sandwich enzyme-linked immunosorbent assay with monoclonal antibodies against proteasome 20Sα6 subunit as catching antibody and polyclonal antibodies against complement factor 3 (*2A*) or complement factor 4 (*2B*) as detecting antibody. The infected rats showed significantly higher values than controls (*p* < 0.001)

The proteasomes in CSF were bound to both C3 (Fig. 2a) and C4 (Fig. 2b) and showed a pronounced increase in both cases, with test/control ratios of 20 (*p* < 0.001) and 7 (*p* < 0.001), respectively. Thus, there was a concomitant aggregation of C3 and C4 with proteasomes. These findings suggest a co-localization of C3 and C4 in the compleasome. Interestingly, both C3 and C4 are involved in the recently discovered degradation of viral particles by intracellular proteasomes (Tam et al. 2014). The deposition and covalent attachment of complement factors onto pathogens results in its translocation into cells during infection, in which it simultaneously induces an antiviral state and directs the degradation of virus particles. This finding suggests that also the extracellular interaction between C3, C4 and proteasomes is initially involved in virus degradation.

The complement factors are produced locally in the brain, and during HSV-1 infection a general complement activation is achieved intrathecally (Orsini et al. 2014, Eriksson et al. 2016). This activation probably contributes to the high intracranial pressure during encephalitis. Intranasal administration of the proteasomal AF-16 peptide has been shown to decrease intracranial pressure, completely abolishing the development of clinical symptoms in the rat model (Jennische et al. 2008). The mechanism of this action is unknown but is probably connected to the formation of compleasomes.

### In vitro test of compleasome formation

In order to study the formation of compleasomes in vitro, proteasomes and complement factors were incubated in a physiological phosphate buffer containing ATP. As seen in Table 1, the proteasomes alone gave no significant response. In contrast, the proteasomes and complement factors together gave a response both with 26S proteasomes and with the catalytic portion 20S. The fact that controls with albumin or IgG antibodies gave no reaction suggests that the proteasomes catalysed the reaction rather than acting as passive recipients of complement factors.

**Table 1:** In vitro test of compleasome reactions

	C3	C4
26S	0	0
26S + C3	0.09	0
26S + C3 + C4	0.30	0.81
20S	0	0
20S + C3 + C4	0.28	0.63

Incubation of 26S proteasomes and complement factors 3 and 4 (C3 and C4) for 3 h at 37 °C. The formation of compleasome complexes was assayed by sandwich enzyme-linked immunosorbent assay using 20Sα6 as catching antibody and anti C3 or C4 as detecting antibody. All proteins were in equimolar concentration (20 nM). The values given here are absorbance at 405 nm at 1/3 dilution

The role of extracellular proteasomes is not known. However, they are enzymatically active in CSF, which suggests that they are able to remove defective proteins in the same way as in cells (Mueller et al. 2012). The virus ring finger protein ICP0 binds to proteasomes and manipulates their degradation of proteins and might be involved in the compleasome formation. Our previous studies showed that a specific part of the proteasome subunit AF/RPN10 inhibited the lethal intracranial pressure during HSE (Jennische et al. 2008). The AF protein is exposed and possibly released during degradation of compleasomes (Lonnroth et al. 2016). This degradation was not achieved in the present study, but might occur during the later course of the infection. The HSV-1 induced rise of proteasomes and their reaction with complement factors imply that they are a part of the innate immune system. In conclusion, HSV-1 induces complex formation of proteasomes and complement factors 3 and 4 in a reaction which could be mimicked in vitro. Whether this response is beneficial or destructive remains to be seen.
